# Risk of Developing Depressive Disorders following Rheumatoid Arthritis: A Nationwide Population-Based Study

**DOI:** 10.1371/journal.pone.0107791

**Published:** 2014-09-16

**Authors:** Shu-Li Wang, Cheng-Ho Chang, Li-Yu Hu, Shih-Jen Tsai, Albert C. Yang, Zi-Hong You

**Affiliations:** 1 Department of Psychiatry, Kaohsiung Veterans General Hospital, Kaohsiung, Taiwan; 2 Department of Dental Laboratory Technology, Central Taiwan University of Science and Technology, Taichung, Taiwan; 3 Department of Psychiatry, Taipei Veterans General Hospital, Taipei, Taiwan; 4 School of Medicine, National Yang-Ming University, Taipei, Taiwan; 5 Center for Dynamical Biomarkers and Translational Medicine, National Central University, Chungli, Taiwan; 6 Division of Nephrology, Department of Internal Medicine, Taichung Veterans General Hospital, Chia-Yi Branch, Chia-Yi, Taiwan; 7 Institute of Medicine, Chung Shan Medical University, Taichung, Taiwan; University of Michigan, United States of America

## Abstract

**Background & Aims:**

To evaluate the risk of depressive disorders among rheumatoid arthritis (RA) by using the Taiwan National Health Insurance Research Database (NHIRD).

**Methods:**

We conducted a retrospective study of a matched cohort of 18 285 participants (3 657 RA patients and 14 628 control patients) who were selected from the NHIRD. Patients were observed for a maximum of 10 years to determine the rates of newly diagnosed depressive disorders, and Cox regression was used to identify the risk factors associated with depressive disorders in RA patients.

**Results:**

During the 10-year follow-up period, 205 (11.2 per 1000 person-years) RA patients and 384 (5.1 per 1000 person-years) control patients were diagnosed with depressive disorders. In RA patients, most depressive disorders (n = 163, 80%) developed with five years of being diagnosed with RA. The incidence risk ratio of depressive disorders between RA patients and control patients was 2.20 (95% confidence interval [CI], 1.84–2.61, *P*<.001). After adjusting for age, sex, and comorbidities, RA patients were 2.06 times more likely to develop depressive disorders (95% CI, 1.73–2.44, *P*<.001) compared with the control patients. Hyperthyroidism (HR = 1.67) was an independent risk factor for depressive disorders in patients with RA.

**Conclusions:**

The likelihood of developing depressive disorders is greater among RA patients than among patients without RA. Symptoms of depression should be sought in patients with RA.

## Introduction

Rheumatoid arthritis (RA) is a systemic chronic autoimmune disorder affecting primarily cartilage and bone. In addition, several organs such as lung, vessels, and kidney may be involved. In general, RA affects 0.5–1% of the population [1]. Despite of substantial advances in medical treatment, chronic pain, fatigue, and functional disability causes much psychosocial burden for these patients with RA [2], [3].

Therefore, interest in the psychiatric aspects of rheumatoid arthritis has grown. Several studies have reported a high depression prevalence rate in patients with RA [4]–[12], and a significant correlation was observed among depression and increased pain levels in patients with RA [13]. Comorbid depression has been also shown to independently increase disability and a low quality of life in patients with RA [14]–[18]. In addition, depression has been demonstrated to be an independent risk factor for non-suicide mortality in patients with RA [19]. Hence, the depression symptoms should be examined in patients with RA because depression is a modifiable illness that is amenable to treatment.

However, in previous studies focusing on depression among patients with RA, depression was often evaluated by using rating scales, such as the Beck Depression Inventory or the Epidemiological Studies Depression Scale, rather than diagnosis by a psychiatrist [5]–[12]. The real prevalence rate of clinical depressive disorders in patients with RA remains unknown. In addition, the design of former studies prevented the temporal causal relationship between RA and depressive disorders from being clarified because the designs of these studies were almost cross-sectional studies [4]–[12]. Furthermore, whether there is an independent risk factor for newly diagnosed depressive disorders in patients with RA has never been studied.

We performed a population-based retrospective cohort study using data derived from the National Health Insurance (NHI) system in Taiwan. The purpose of our study was to determine whether RA was associated with an increased risk of clinical depressive disorders. Independent risk factors for newly diagnosed depressive disorders among the patients with RA were also identified.

## Materials and Methods

### Data Sources

Instituted in 1995, the NHI program is a mandatory health insurance program that offers comprehensive medical care coverage, including outpatient, inpatient, emergency, and traditional Chinese medicine, to all residents of Taiwan; the coverage rate is as high as 99% [20]. The NHI research database (NHIRD) contains comprehensive information regarding clinical visits, including prescription details and diagnostic codes based on the International Classification of Diseases, ninth revision, Clinical Modification (ICD-9-CM). The NHIRD is managed by the National Health Research Institutes (NHRI) and confidentiality is maintained according to the directives of the Bureau of the NHI. The data source for our study was the Longitudinal Health Insurance Database 2005 (LHID 2005), which is a dataset of NHIRD. Data for the LHID was collected by systematically and randomly sampling from the NHIRD; the database included the data of one million individuals. The NHRI of Taiwan reports that there were no significant differences in gender distribution, age distribution, or average insured payroll-related amount between the patients in the LHID and those in the original NHIRD [21]. Detailed information about the data source is provided on the NHRI website (http://nhird.nhri.org.tw). Comments, problems or requests about the data application could be sent to e-mail address at the NHRI (e-mail: nhird@nhri.org.tw).

### Ethics Statement

The Institutional Review Board of the Taipei Veterans General Hospital approved this study (2013-03-035AC). Written consent from study patients was not obtained because the NHI dataset consists of de-identified secondary data for research purposes, and the Institutional Review Board of Taipei Veterans General Hospital issued a formal written waiver for the need for consent.

### Study Population

Using data extracted from the LHID 2005, we conducted a retrospective cohort study of patients aged 20 years and older who were newly diagnosed with RA by a rheumatologist between January 1, 2000 and December 31, 2008. RA was defined as ICD-9-CM code: 714. We excluded patients who were diagnosed with depressive disorders (ICD-9-CM code: 296.2X-296.3X, 300.4, and 311.X) before enrollment. Insurance premiums, calculated according to the beneficiary's total income, were used to estimate monthly income. Monthly income was grouped into low income (monthly income <20,000 New Taiwan Dollar [NTD]), median income (20,000 NTD≤monthly income <40,000 NTD), and high income (monthly income ≥40,000 NTD). Urbanization was divided into three groups: urban, suburban, and rural. Urbanization and monthly income levels were used to represent socioeconomic status. For each cirrhotic patient included in the final cohort, 4 age-, sex-, and enrolment-date-matched control patients who were not diagnosed with rheumatoid arthritis or depressive disorder were randomly selected from the LHID 2005. All RA and control patients were observed until diagnosed with a depressive disorder by a psychiatrist, death, withdrawal from the NHI system, or December 31, 2009. The primary clinical outcome assessed was psychiatrist-diagnosed depressive disorder.

### Statistical Analyses

The incidence of newly diagnosed depressive disorders in the RA and control patients, stratified by gender and age (equal or older than 60 years old or younger than 60 years old) was calculated. Independent *t*-tests and chi-squared tests were used to examine the differences in the demographic characteristics between the RA and control patients.

A Cox proportional-hazards regression model was used to identify variables that predicted depressive disorder in the RA and control patients, and in the RA patients only. Control variables, such as age; sex; common comorbidities, including hypertension, diabetes mellitus, dyslipidemia, coronary artery disease, congestive heart failure, cirrhosis, hyperthyroidism, hypothyroidism, cerebrovascular disease, and malignancy; urbanization; and monthly income were included as covariates in the univariate model. Factors that demonstrated a moderately significant statistical relationship in the univariate analysis (*P*<.1) were entered by forward selection in a multivariate Cox proportional-hazards regression model [22].

The Perl programming language (version 5.12.2) was used to extract and compute data. The Microsoft SQL Server 2005 (Microsoft Corp., Redmond, WA, USA) was used for data linkage, processing, and control sampling. IBM SPSS (version 19.0 for Windows; IBM Corp., New York, NY, USA) and SAS statistical software (version 9.2; SAS Institute Inc., Cary, NC, USA) were used to perform all statistical analyses. The comparisons resulting in a *P*-value of less than .05 were considered to indicate a statistically significant relationship.

## Results

### Participant Selection (Figure 1)

The sample comprised 3657 RA patients and 14 628 control patients without depression, among whom 72.8% were female. The median age at enrollment was 51 years (interquartile range [IQR], 41–61 years), and the median follow-up period was 4.72 years (IQR, 2.59–7.42 years) for RA patients and 4.92 years (IQR, 2.69.21–7.55 years) for control patients. The comorbidities, including hypertension, diabetes mellitus, dyslipidemia, congestive heart failure, hyperthyroidism, hypothyroidism, and cerebrovascular disease were more common in RA patients than control patients. The comparisons of the demographic and clinical variables between the RA and control patients are presented in Table 1.

**Table 1:** Baseline Characteristics of Patients with and without Rheumatoid Arthritis.

Demographic data	Patients with rheumatoid arthritis		Patients without rheumatoid arthritis		*P*-value
	*n* = 3,657		*n* = 14,628		
	*n*	%	*n*	%	
Age (years) (interquartile range)	51 (41–61)		51 (41–61)		
≥60	961	26.3	3,851	26.3	.966
<60	2,696	73.7	10,777	73.7	.
Sex					
Male	993	27.2	3972	27.2	.999
Female	2,664	72.8	10,656	72.8	.
Comorbidities					
Hypertension	1,186	32.4	4,032	27.6	<.001
Diabetes mellitus	687	18.8	2,305	15.8	<.001
Dyslipidemia	1,098	30.0	3,002	20.5	<.001
Coronary artery disease	45	1.2	168	1.1	.673
Congestive heart failure	172	4.7	533	3.6	.004
Cirrhosis	62	1.0	198	1.4	.117
Hyperthyroidism	219	6.0	477	3.3	<.001
Hypothyroidism	107	2.9	178	1.2	<.001
Cerebrovascular disease	316	8.6	929	6.4	<.001
Malignant neoplasms	96	2.6	338	2.3	.274
Degree of urbanization					
urban	2,238	61.2	8,787	60.1	.435
suburban	1,040	28.4	4,268	29.2	
rural	326	8.9	1,362	9.3	
Income group					
low income	1,731	47.3	7,151	48.9	.001
median income	1,430	39.1	5,820	39.8	
high income	496	13.6	1,657	11.3	
Follow-up years (median)	4.72 (2.59–7.42)		4.92 (2.69–7.55)		.004

**Figure 1:**
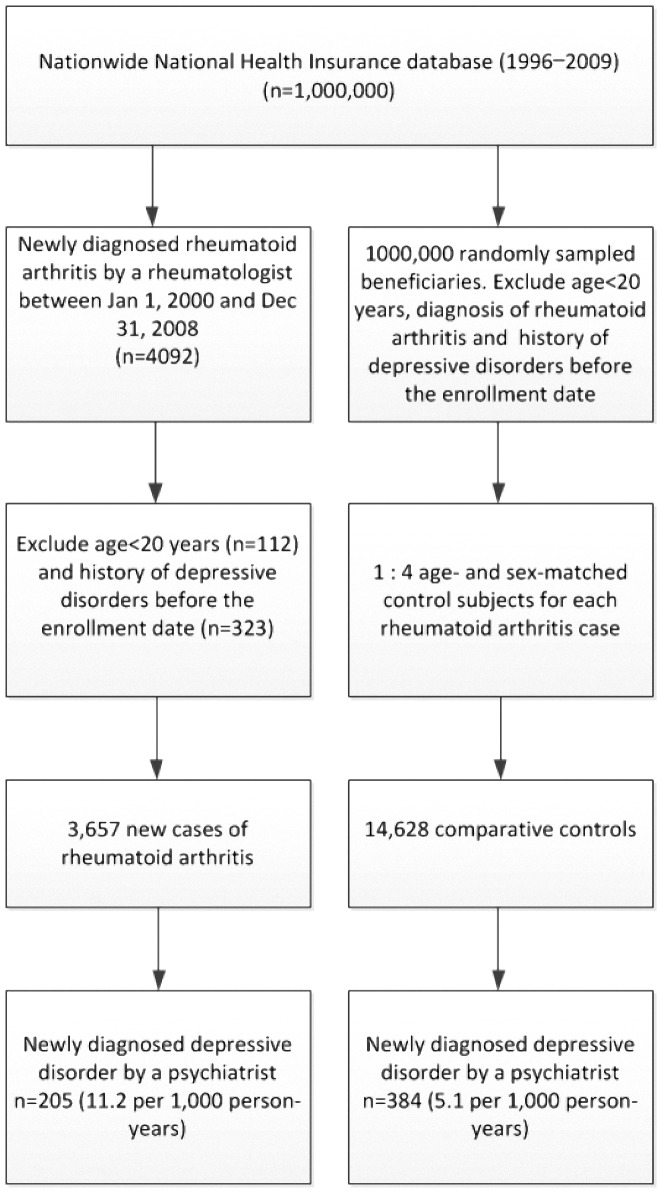
Flow chart of sample selection criteria.

### Incidence Rate of Depressive Disorders

During the study period, 205 (11.2 per 1000 person-years) RA patients and 384 (5.1 per 1000 person-years) control patients were diagnosed with depressive disorders. The incidence risk ratio (IRR) of depressive disorders between RA patients and control patients was 2.20 (95% CI, 1.84–2.61, *P*<.001). When stratified by gender and age, the IRR of depressive disorders was still higher in the RA patients than in the control patients. The results are shown in Table 2. In RA patients, most depressive disorders (n = 163, 80%) developed with five years of being diagnosed with RA.

**Table 2:** Incidence of Depressive Disorders in Patients with and without Rheumatoid Arthritis.

	Patients with rheumatoid arthritis		Patients without rheumatoid arthritis		Risk ratio (95% CI)	*P* value
	No. of Depressive disorders	Per 1 000 person-years	No. of Depressive disorders	Per 1 000 person-years		
Total	205	11.2	384	5.1	2.20 (1.84–2.61)	<.001
Age						
≥60	49	2.8	109	1.4	1.85 (1.29–2.61)	<.001
<60	156	8.5	275	3.7	2.33 (1.91–2.85)	<.001
Sex						
Male	45	2.5	88	1.2	2.10 (1.44–3.05)	<.001
Female	160	8.7	296	3.9	2.22 (1.82–2.70)	<.001

CI indicates confidence interval.

### Rheumatoid arthritis on Risks of Clinical Depression

**Figure 2:**
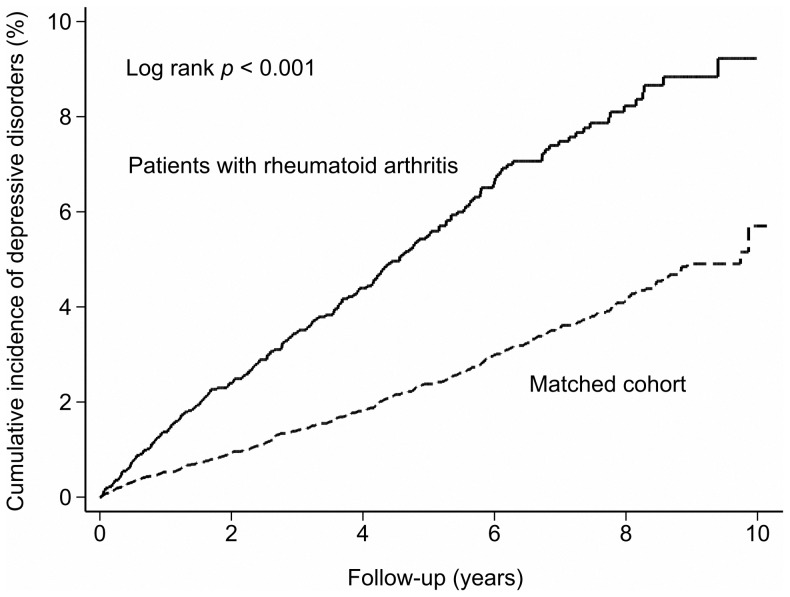
Cumulative incidence of depressive disorders in patients with rheumatoid arthritis (RA) and matched cohort.

After adjusting for age, sex, comorbidities, urbanization, and monthly income, the hazard ratio (HR) for developing depressive disorders during the follow-up period was 2.06 times (95% CI, 1.73–2.44, *P*<.001) greater for the RA patients than for the control patients (Table 3 and figure 2).

**Table 3:** Analyses of Risk Factors for Depressive Disorders in Patients with and without Rheumatoid Arthritis.

Predictive variables	Univariate analysis		Multivariate analysis	
	HR (95% CI)	*P* value	HR (95% CI)	*P* value
Rheumatoid arthritis	2.20 (1.86–2.60)	<.001	2.06 (1.73–2.44)	<.001
Age (<60 = 1, ≥60 = 0)	0.91 (0.76–1.10)	.327		
Sex (female = 1, male = 0)	1.34 (1.10–1.62)	.003	1.29 (1.06–1.57)	.011
Comorbidities				
Hypertension	1.39 (1.17–1.65)	<.001	1.00 (0.82–1.21)	.957
Diabetes mellitus	1.63 (1.34–1.99)	<.001	1.20 (0.96–1.50)	.116
Dyslipidemia	1.77 (1.48–2.12)	<.001	1.39 (1.14–1.71)	.001
Coronary artery disease	1.32 (0.62–2.77)	<.001	1.00 (0.47–2.13)	.995
Congestive heart failure	1.16 (0.75–1.79)	.505		
Cirrhosis	2.33 (1.39–3.89)	.001	1.91 (1.14–3.20)	.014
Hyperthyroidism	2.24 (1.61–3.12)	<.001	1.79 (1.27–2.52)	.001
Hypothyroidism	1.75 (1.01–1.76)	<.001	1.01 (0.57–1.78)	.973
Cerebrovascular disease	2.13 (1.65–2.76)	<.001	1.78 (1.36–2.33)	<.001
Malignant neoplasms	1.16 (0.65–2.05)	.622		
Degree of urbanization				
urban	Reference			
suburban	0.91 (0.76–1.10)	.319		
rural	0.85 (0.63–1.14)	.286		
Income group				
low income	Reference		Reference	
median income	0.94 (0.79–1.12)	.484	0.98 (0.83–1.17)	.847
high income	0.69 (0.51–0.92)	.013	0.75 (0.56–1.02)	.063

HR indicates hazard ratio; CI indicates confidence interval.

### Risks Factors for Depression in Rheumatoid arthritis Patients

In Table 4, we applied univariate analysis to predict the development of depressive disorders in the RA cohort, and the results demonstrated that the female sex (HR = 1.37, 95% CI, 0.98–1.90, *P* = .063), diabetes mellitus (HR = 1.39, 95% CI, 1.00–1.94, *P* = .051), hyperthyroidism (HR = 1.79, 95% CI, 1.09–2.94, *P* = .022), cerebrovascular disease (HR = 1.55, 95% CI, 0.99–2.41, *P* = .054) and high income (HR = 0.56, 95% CI, 0.34–0.94, *P* = .028) are significant prognostic factors. The multivariate analysis confirmed that hyperthyroidism (HR = 1.67, 95% CI, 1.01–2.76, *P* = .045) was an independent risk factor for depressive disorder in the patients with RA.

**Table 4:** Analyses of Risk factors for Depressive Disorders in Rheumatoid Arthritis Patients.

Predictive variables	Univariate analysis		Multivariate analysis	
	HR (95% CI)	*P* value	HR (95% CI)	*P* value
Age (<60 = 1, ≥60 = 0)	1.07 (0.77–1.47)	.701		
Sex (female = 1, male = 0)	1.37 (0.98–1.90)	.063	1.28 (0.92–1.79)	.146
Comorbidities				
Hypertension	1.13 (0.84–1.51)	.429		
Diabetes mellitus	1.39 (1.00–1.94)	.051	1.27 (0.90–1.78)	.171
Dyslipidemia	1.23 (0.91–1.66)	.174		
Coronary artery disease	0.97 (0.24–3.92)	.968		
Congestive heart failure	1.11 (0.57–2.16)	.766		
Cirrhosis	1.90 (0.85–4.29)	.120		
Hyperthyroidism	1.79 (1.09–2.94)	.022	1.67 (1.01–2.76)	.045
Hypothyroidism	1.34 (0.63–2.84)	.452		
Cerebrovascular disease	1.55 (0.99–2.41)	.054	1.46 (0.93–2.29)	.098
Malignant neoplasms	1.04 (0.43–2.54)	.925		
Degree of urbanization				
urban	Reference			
suburban	0.96 (0.71–1.31)	.793		
rural	0.78 (0.46–1.33)	.365		
Income group				
low income	reference		reference	
median income	1.04 (0.78–1.38)	.808	1.08 (0.81–1.44)	.603
high income	0.56 (0.34–0.94)	.028	0.62 (0.37–1.04)	.068

HR indicates hazard ratio; CI indicates confidence interval.

## Discussion

The key findings of our study are that (1) the incidence rate of depressive disorders in patients with RA was 11.2 per 1000 person-year; (2) the risk of clinical depressive disorders was higher when patients with RA (HR = 2.06) and most depressive disorders (n = 163, 80%) developed within five years of being diagnosed with RA; (3) hyperthyroidism (HR = 1.67) might be an independent risk factor for depressive disorders among patients with RA.

The strengths of this study are the large sample size, the long follow-up period, and the diagnosis of clinical depressive disorders and rheumatoid arthritis by specialists. In addition, our study design involved an unbiased participant selection process. Because participation in the NHI is mandatory and all residents of Taiwan can access low-cost health care, referral bias is low and follow-up compliance is high.

Consistent with the results of previous studies [4]–[11], we found that the risk of depression in RA patients was higher than that in the non-RA control patients. Chronic inflammation is one of the possible causes. RA is a chronic inflammatory disease, and accumulating evidence implicates inflammation as a critical mediator in the pathophysiology of depressive disorders [23]–[26]. In the Cardiovascular Health Study of 4 268 patients, a significant association was noted between high levels of C-reactive protein and depression symptoms, even after adjusting for confounding factors [27]. Administration of cytokines including tumor necrosis factor-α in animal and human studies has been shown to induce depressive symptoms, including sleep disturbance, anorexia, loss of interest and mood disturbances [28]. Potential involved mechanisms include direct effects of pro-inflammatory cytokines on monoamine, dysregulation of the hypothalamic-pituitary-adrenal axis, impaired neuroplasticity and structural and functional brain changes [29].

The symptoms of RA such as pain and fatigue may be another possible cause for higher risk of depression in RA patients than that in the non-RA control patients. Symptoms of RA such as pain and fatigue may impair patients' function and quality of life and influence patients' perception of health status, and make the patients more prone to develop depressive symptoms [12], [30]. RA patients often suffered pain and pain intensity was more clearly related to disability in RA patients [31]. Several studies have shown that chronic pain was an important risk factor for depression and suicidal behavior [32]–[34]. Previous study has shown that the degree of depression in RA patients varies in proportion to the level of pain [31].

Furthermore, physical comorbidities might be another possible cause for a higher prevalence of depressive disorder in RA patients. Our findings are consistent with the results of previous studies that found that a higher percentage of many physical comorbidities in RA patients, such as hypertension [35], diabetes mellitus [36], congestive heart failure [37], hypothyroidism [38], and cerebrovascular disease [39]. All of above diseases had been proposed to be related with increased the risk of depression [40]–[43].

Finally, long-term drug use in RA patients and inevitable side effects of various medications might also impaired quality of life in RA patients and increased the risk of depression in RA patients [44], [45]. Previous study showed that disease-modifying drugs used for RA patients had been related to higher rates of depression, and suicidal ideation among RA patients, serving as an alert to the importance of considering also this factors in therapeutic decisions [46].

According to previous studies, conservative estimates of the prevalence of depressive disorder among patients with RA ranged between 13 and 20% [13], [45], [47]. This prevalence rate was much higher than the rate calculated in this study. The possible reason for these two discrete findings might be partly due to difference in the methods of assessing depression [13]. In the most previous studies regarding RA and depression [5]–[12], rating scales, such as the Beck depression inventory, rather than diagnosis by a psychiatrist, were used for evaluation of depression in those studies. People who were recorded as depressed on rating scales may not meet the diagnostic criteria for clinical depressive disorders. However, depressive disorders in RA patients may be underdiagnosed in our study because symptoms of depressive disorders may mimic symptoms of RA, such as easily fatigue, sleep disturbance, and loss of interests with multiple somatic complaints. Physicians may have viewed RA patients' depressive symptoms as physical discomforts caused by RA and overlooked these symptoms, rather than viewing these symptoms as warning signs of depressive disorder.

There has been some discussion in the literature regarding the factors that influence the development of depression in patients with RA [48]–[50]. However, whether there is certain physical comorbidity as an independent risk factor for new-onset depressive disorders following a RA diagnosis has never been studied. In our study, we found that hyperthyroidism was an independent risk factor for depressive disorders in patients with RA. Studies have shown that mood disturbance, especially depressive disorders, often accompanies hyperthyroidism patients [51]–[54]. Alterations in components of the hypothalamic-pituitary-thyroid axis have been documented for the development of depressive disorder among patients with hyperthyroidism [53]. In addition, functional impairment caused by hyperthyroidism might play a vital role in causing psychological distresses including depressive disorders among patients with hyperthyroidism [55].

Our findings have certain limitations. Information regarding the family history of psychiatric disorders [56], [57], lifestyle factors [58], self-perceived health, disability [59], and environmental factors (life stresses, such as recent major life event, domestic violence, childhood trauma, and social support) [60]–[63] are not included in the NHIRD, and all of which might be associated with the risk of development of depressive disorders. In addition, the severity of RA could not be ascertained. Whether the severity of RA influences the risk of developing depressive disorders warrants further study, despite previous studies indicating that disease activity of RA alone does not equate with depressive symptoms in RA patients reliably [47]. Furthermore, physicians may view RA patients' depressive symptoms as physical discomforts caused by RA and overlook these symptoms. Therefore, in our study, the incidence of clinical depressive disorders may be underestimated.

This population-based retrospective cohort study demonstrated that the risk of developing depressive disorders is higher among RA patients than those without RA, and that RA is an independent risk factor for depressive disorders. Hyperthyroidism is an independent risk factor for developing depressive disorders among patients with RA. Because depression was related to impairing the quality of life of RA patients, and depressive disorder is modifiable illness that is amenable to treatment, symptoms of depression should be sought in patients with RA. Future population-based prospective studies are needed to further investigate the association between RA and the risk of developing depressive disorders.
